# Therapeutic Application and Structural Features of Adeno-Associated Virus Vector

**DOI:** 10.3390/cimb46080499

**Published:** 2024-08-02

**Authors:** Yasunari Matsuzaka, Ryu Yashiro

**Affiliations:** 1 Division of Molecular and Medical Genetics, Center for Gene and Cell Therapy, The Institute of Medical Science, The University of Tokyo, Minato-ku, Tokyo 108-8639, Japan; 2 Administrative Section of Radiation Protection, National Institute of Neuroscience, National Center of Neurology and Psychiatry, Kodaira 187-8551, Japan; ryuy@niid.go.jp; 3 Department of Mycobacteriology, Leprosy Research Center, National Institute of Infectious Diseases, Tokyo 162-8640, Japan

**Keywords:** adeno-associated virus, capsid screening, empty particles, viral packaging

## Abstract

Adeno-associated virus (AAV) is characterized by non-pathogenicity, long-term infection, and broad tropism and is actively developed as a vector virus for gene therapy products. AAV is classified into more than 100 serotypes based on differences in the amino acid sequence of the capsid protein. Endocytosis involves the uptake of viral particles by AAV and accessory receptors during AAV infection. After entry into the cell, they are transported to the nucleus through the nuclear pore complex. AAVs mainly use proteoglycans as receptors to enter cells, but the types of sugar chains in proteoglycans that have binding ability are different. Therefore, it is necessary to properly evaluate the primary structure of receptor proteins, such as amino acid sequences and post-translational modifications, including glycosylation, and the higher-order structure of proteins, such as the folding of the entire capsid structure and the three-dimensional (3D) structure of functional domains, to ensure the efficacy and safety of biopharmaceuticals. To further enhance safety, it is necessary to further improve the efficiency of gene transfer into target cells, reduce the amount of vector administered, and prevent infection of non-target cells.

## 1. Introduction

From the perspective of application as a vector for gene therapy, AAV (adeno-associated virus) is one of the gene transfer vectors expected to be used for non-pathogenic and safe gene therapy [1,2]. Wild-type AAV consists of non-enveloped icosahedral virus particles with a diameter of approximately 23 to 28 nm and lacks essential lipids, carbohydrates, accessory proteins, and histones. As a result, AAV has low immunogenicity, and few antibodies are produced. Therefore, antibody-dependent cytotoxicity is not observed. On the other hand, there is also the disadvantage that the humoral immune system is activated by the viral infection. The benefit of AAV is limited in some applications because it is neutralized by the immune system. In addition, AAV vectors can efficiently deliver genes into non-dividing cells, and gene expression can persist for long periods of time. This AAV is a non-enveloped virus belonging to the genus Dependoparvovirus in the family Parvoviridae [3]. In other words, it is an approximately 23 to 28 nm, helper virus (adenovirus)-dependent, non-enveloped virus that infects humans and primates. Ying Yang1 (YY1) repression of the p5 promoter limits the expression of Rep68/Rep78 when adenoviruses or other similar helper viruses are not simultaneously infecting the host. p5 promoter repression inhibits AAV genome replication and gene expression, leading to AAV chromosomal integration, specifically at a mapped site called adeno-associated virus integration site 1 (AAVS1) on human chromosome 19. Wild-type AAV is the only eukaryotic virus that integrates site-specifically into the human genome. AAV particles have a structure in which approximately 4.7 kb of linear single-stranded DNA is encapsulated in an icosahedral capsid [4].

**Figure 1:**
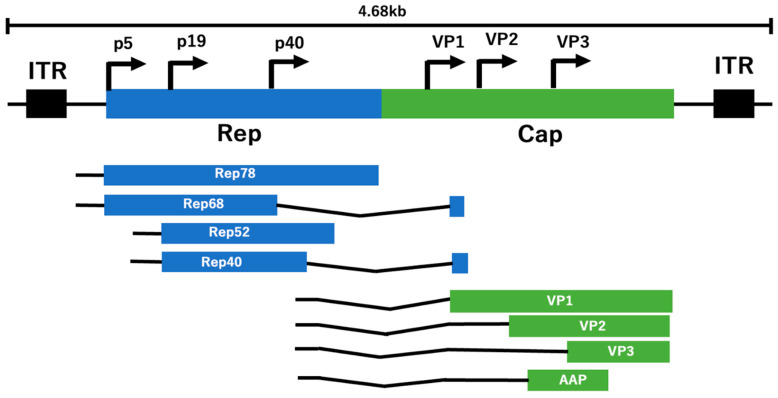
Structure of the wild-type AAV genome. Rep78 and Rep68 are expressed from the p5 promoter and Rep52 and Rep40 from the p19 promoter. VP1, 2, 3 and the assembly-activating protein (AAP) are translated from the p40 transcript encoded by the cap gene.

AAV can deliver its genome to both mitotic and non-mitotic cells and can survive outside the chromosomes without integrating its genome into the host cell. The viral genome of wild-type AAV contains a 145-base hairpin-structured inverted terminal repeat (ITR) sequence. This sequence is necessary for transcription, polyadenylation, amplification, and insertion of the positive-strand RNA viral genome into the host genome by integrase at both ends. In addition, there are three genes, including the replicase (Rep) gene, which produces four non-structural proteins: Rep78, Rep68, Rep52, and Rep40; the capsid (Cap) gene, which contains three types of viral proteins (VPs: VP1, VP2, and VP3) that make up the AAV capsid; and the small cofactor gene, which encodes an assembly-activating protein, are encoded between these ITR sequences. Since this region serves as the replication initiation site, DNA synthesis can be performed without DNA polymerases (self-priming) (Figure 1) [5,6,7]. When recombinant AAV is used for research purposes, the Rep protein is delivered in trans, eliminating the ability of recombinant AAV (rAAV) to integrate into its preferred site of genomic integration on human chromosome 19, termed adeno-associated virus integration site 1 (AAVS1). Thus, the function of the Rep protein in gene integration is closely linked to its role in viral DNA replication and regulation of AAV gene expression. The review highlights the importance of evaluating engineered capsids and structural features for clinical applications. The structural studies highlighted specific capsid regions, residues and properties such as surface topology, charge distribution, and antibody binding that influence AAV tropism and could guide the rational engineering of AAV vectors for targeted gene delivery. Various biophysical and structural techniques, such as confined proteolysis, cryo-EM, mutagenesis, and structural analysis, have been used to characterize the conformational dynamics of AAV capsid proteins and their functional implications in processes such as cell entry, genome packaging, capsid assembly, and viral trafficking [8]. Hydrogen/deuterium exchange–mass spectrometry is a powerful tool for characterizing the conformational dynamics and functions of capsid proteins, providing valuable insights into their structure, dynamics, and interactions, which are essential for understanding viral life cycles and developing antiviral strategies [9,10,11]. However, there are several other complementary methods that can provide similar insights into the dynamics and functions of AAV capsid proteins: (i) limited proteolysis and peptide mapping, (ii) differential scanning calorimetry (DSSC) and fluorimetry, (iii) electron microscopy, (iv) native mass spectrometry, etc. [12,13]. In this review, we summarize the structure, tropism, and modification of viral capsids in AAV due to the development of efficient and safe gene therapy vectors.

## 2. Genes and Genomic Structure of AAV

In addition, the membrane-associated accessory protein (MAAP) is located in the same genomic region as the unique VP1/2 domain. However, a different reading frame is encoded as the fourth open reading frame (ORF). Therefore, large genes are not suitable for use with AAV vectors. Rep78 and Rep68 mRNAs are transcribed from the p5 promoter and Rep52 and Rep40 mRNAs from the p19 promoter. On the other hand, Cap mRNA is transcribed from the p40 promoter [14,15]. Rep78 and Rep68 proteins bind to calmodulin in the presence of calcium ions. During this process, transcription of the Cap gene in AAV is upregulated by the p40 promoter and downregulated by the p5 and p19 promoters. This step has low efficiency and is the rate-limiting step in the infection process. Increasing the efficiency of this step could potentially have more significant effects at an earlier stage of infection [16,17]. Studies have shown that modifications in the Rep and Cap genes, such as splitting them into two different plasmids or using chimeric Rep genes, can increase the efficiency of AAV production by increasing the proportion of total capsids and improving transduction efficiency [18,19,20].

In addition, modifications such as separating the Rep nicking site from the Rep binding site in the P5 promoter can reduce contaminants and improve the use of AAV vectors in gene therapy [19]. The Rep protein has roles such as regulation of AAV promoter activity, replication of the viral genome, packaging it into the capsid, and integration of the viral genome into the chromosome of the host cell, which has been termed AAVS1 on chromosome 19 at a low integration rate [21]. This feature is superior to retroviruses, which cause random gene insertion and subsequent mutations and cancerous transformation of cells.

Most AAV gene insertions occur in AAVS1, and integration is now accepted to be more than negligible, estimated to be above 3% [22]. However, removal of the Rep and Cap genes also removes the ability of this gene to insert. The large Rep78/68 has activities such as endonuclease, helicase, and ATPase [23]. In addition, the small Rep (Rep52/40) is required for the accumulation of single-stranded genomic DNA [24]. The ITR itself forms Watson–Crick base pairs and T-shaped hairpin structures containing cis-elements necessary for replication and packaging. This portion of the ITR serves as an initiation point for replication, acts as a primer, and is also required for packaging into viral particles and integration into the chromosomal DNA of host cells [6,25]. Furthermore, Rep protein is only expressed in AAV-producing cells or cells infected with wild-type AAV and is not incorporated into AAV capsids. All Rep proteins have helicase activity and are essential for initiation and integration into the host cell chromosome. The AAV capsid has an icosahedral structure in which 60 VPs are associated [13,26]. The VP composition ratio of the AAV2 capsid is estimated to be VP1 (82 kDa)/VP2 (65 kDa)/VP3 (60 kDa) = 1:1:10 in terms of molar ratio, but individual capsid composition is divergent and stochastic due to mosaicism [4,13,27]. The diameter of AAV particles is about 20 to 25 nm. And the molecular weight is about 5.3 MDa including DNA [28,29].

## 3. Serotype of AAVs

**Figure 2:**
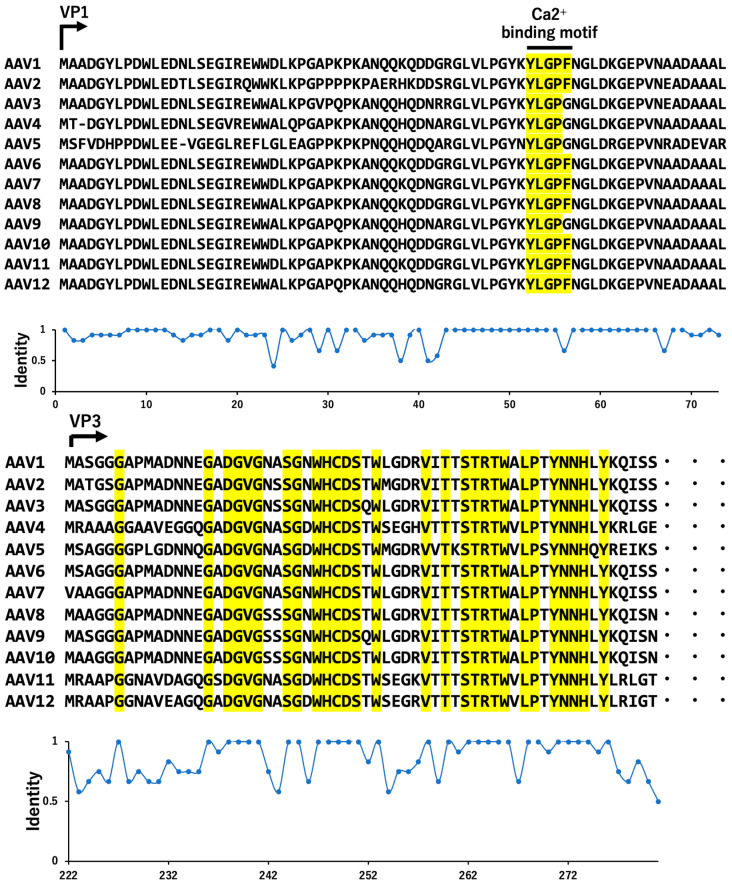

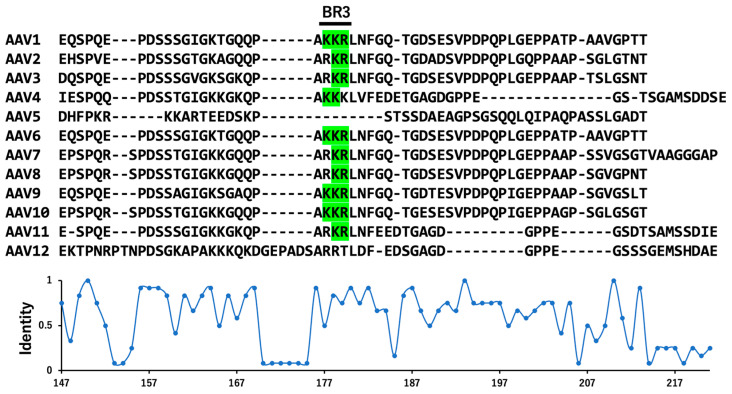
**(Upper)** Amino acid sequence in VP1 and VP2 of AAV type 1 to type 12. (**Bottom**) Amino acid identity of VP1 and VP2 of AAV type 1 to type 12. Yellows highlight the conserved amino acid motif. BR3 domain is indicated by green.

**Figure 3:**
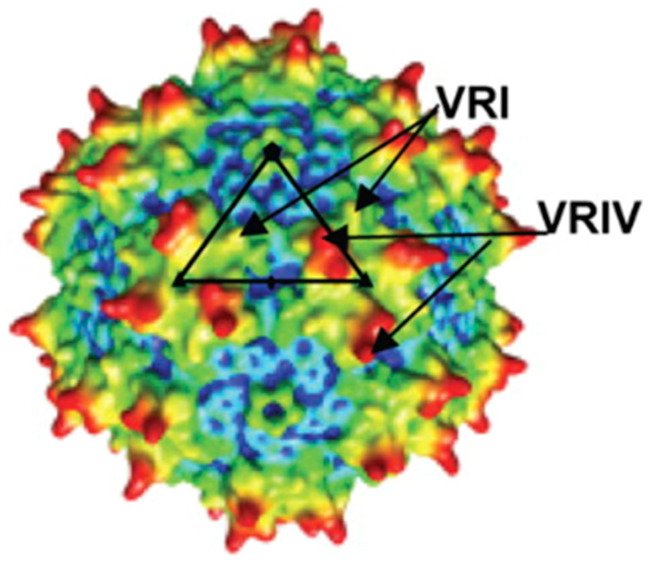
AAV2 structures at a 9.7-Å resolution. Example capsid surface regions corresponding to VR-I and VR-IV are indicated by arrows in panels [45].

Unlike AAV2, AAV1 does not bind heparin due to the absence of specific amino acid residues, which makes its purification different. In addition, studies have shown that AAV1 is highly efficient in transducing skeletal muscle, neurons, glial cells, ependymal cells in the brain, and even the heart [30,31]. In addition, common AAV sequences were found in monkey and human tissue DNA, and serotypes 7 to 9 were proposed. This identification represents the discovery of new AAV variants with distinct genomic sequences and characteristics, expanding the known diversity of AAV serotypes beyond those previously recognized. The research highlights the importance of studying and characterizing these novel AAV serotypes for potential applications in gene therapy and other biomedical fields [1,32,33]. Most AAV capsid proteins are structurally similar, with 80% to 88% homology to type 2 capsid amino acid sequences and 78% to 82% DNA sequence homology (Figure 2). The precise definition of a serotype is that a newly isolated serotype does not efficiently cross-react with neutralizing antibodies specific for all other existing serotypes [34,35,36,37]. Among the major serotypes of AAV, AAV2, AAV3, AAV5, and AAV6 have been discovered in human serum, and other serotypes have been discovered in non-human primate serum [37]. Characteristics of AAV include the types of cell surface receptors to which they bind during cell invasion and the tissue tropism that facilitates the introduction of the genes contained, depending on the serotype (Figure 3) [38,39]. AAVs mainly use proteoglycans as receptors to invade cells, but the types of sugar chains in proteoglycans that have binding capacity differ [40,41,42,43]. Furthermore, there are differences in the tissues into which endogenous genes are easily introduced, such as kidney for AAV2 and liver for AAV3 [30,44].

There are several serotypes of AAVs, such as AAV1, AAV2, etc., which differ in their capsid proteins, resulting in variations in tissue tropism and transduction efficiency. These differences make specific AAV serotypes more suitable for targeting different tissues or cell types in gene therapy applications. The main differences between AAV serotypes lie in their tissue tropism, transduction efficiency, and immunogenicity. For example, AAV1 is an efficient serotype for gene transfer to skeletal muscle, with similar or higher transduction efficiency than other commonly used serotypes such as AAV2, AAV7, and AAV8 [30]. In a study comparing different AAV serotypes (AAV2/1, AAV2/2, AAV2/5, AAV2/7, and AAV2/8) in mouse skeletal muscle, AAV2/1 showed transduction efficiencies similar to those of AAV2/7 and AAV2/8 and higher than those of AAV2/2 and AAV2/5. AAV1 was able to efficiently transduce both slow and fast muscle fibers in mice. In a clinical trial of AAV1-mediated gene transfer to skeletal muscle for lipoprotein lipase deficiency, AAV1 successfully transduced muscle and resulted in T-cell activation against the AAV1 capsid in four out of eight subjects, indicating efficient muscle transduction [46]. AAV1 has a lower affinity for heparin than AAV2, which may contribute to its efficient muscle transduction [47]. However, it is important to note that muscle regeneration may affect AAV1 transduction efficiency. One study showed that AAV1 transcription and transduction capacity is drastically reduced in regenerating dystrophic muscle compared to healthy muscle [48]. This suggests that for muscular dystrophies with ongoing muscle degeneration/regeneration, strategies to overcome the effects of regeneration on AAV transduction may be needed. AAV2 is commonly used for gene delivery to the liver; it was one of the earliest adeno-associated virus (AAV) serotypes used for liver-directed gene delivery but has since been surpassed by other serotypes with higher hepatotropism and transduction efficiency. Early studies showed that AAV2 had hepatic and skeletal muscle tropism, making it a candidate for liver gene therapy [49]. However, AAV8 and AAV9 have higher affinity for hepatocytes compared to AAV2. AAV8 can transduce three to four times more hepatocytes and deliver three to four times more vector genomes per transduced cell than AAV2 [50]. In juvenile mouse livers, a single administration of AAV2/8 vectors resulted in rapid loss of episomal vector genomes within 2 weeks, although stable transgene expression was observed in a small percentage of hepatocytes, suggesting genomic integration. Recent clinical trials in liver diseases such as hemophilia have favored AAV serotypes such as AAV5, AAV8, and AAVhu68 over AAV2 due to their improved hepatotropic and transduction efficiency [50]. While AAV2 was an early vector used for liver gene delivery, it has been largely superseded by other AAV serotypes with superior liver transduction profiles.

Regarding AAV serotypes targeting the central nervous system (CNS), AAV1 has tropism for the CNS, heart, liver, lung, kidney, and eye [30,47]. AAV2 can transduce the CNS, liver, muscle, kidney, eye, and brain [47]. AAV4 shows tropism for the CNS, eye, and heart [47]. AAV5 efficiently targets the CNS, eye, lung, liver, and kidney [44,47]. AAV7 achieves robust transduction in cortical and spinal tissues of the CNS in non-human primates [50]. AAV8 transduces the CNS, heart, liver, skeletal muscle, pancreas, eye, retina, brain, and testis [44,47]. AAV9 has the best cardiac transduction specificity and can transduce the CNS, heart, muscle, liver, kidney, lung, pancreas, retina, brain, and testis with high efficiency [50,51,52]. AAV10 has mild tropism for the CNS in non-human primates [44]. As for AAV serotypes for cardiac targeting, AAV1 is considered one of the most favorable serotypes for specific cardiac transduction [47,53]. AAV6 was identified as one of the most cardiotropic serotypes, predominantly infecting cardiomyocytes [53]. AAV8 is considered one of the most favorable candidates for specific cardiac transduction [50,53]. AAV9 has the best cardiac transduction specificity and stable expression [47,53]. Thus, AAV9 appears to be the most promising serotype for targeting the CNS and heart, followed by AAV1, AAV8, and AAV6, which also show good transduction efficiency for these tissues [30,47,53,54].

In addition, the immunogenicity of different serotypes may affect their long-term efficacy in gene therapy. AAV serotypes have been used in gene therapy for various diseases, including monogenic inherited disorders. For example, AAV-based gene therapy is available for hereditary diseases such as retinal dystrophy and spinal muscular atrophy [51,55]. The choice of AAV serotype depends on the specific therapeutic target and the desired tissue or cell type for gene delivery. The main differences between AAV serotypes lie in their tissue tropism, transduction efficiency, and immunogenicity, which influence their application in gene therapy for targeting different tissues and treating different diseases. Selection of the most appropriate AAV serotype is critical to the success of gene therapy interventions.

## 4. Efficacy of Gene Therapy by AAV

In addition, the efficacy of gene therapy may be significantly improved by using vectors derived from serotypes other than type 2 [52]. For example, in a gene therapy experiment in a mouse model of phenylketonuria, a genetic disease in which the amount of phenylalanine in the blood increases, and one of the diseases called inborn errors of metabolism, when targeting the liver, no response was observed with AAV type 2 vector, but a significant and stable effect was observed with type 5 vector [56]. Furthermore, studies with the more potent type 8 vector showed that the same effect could be achieved with a lower amount of vector. By exploiting this difference in tissue tropism, recombinant AAV vectors can be designed to deliver therapeutic genes to target organs [57,58,59,60]. The interaction between viral attachment proteins and cell surface receptors is essential for the attachment phase of infection and influences the course of viral pathogenesis and replication. In addition, combinatorial recognition of co-receptors may also contribute to cell surface interactions during viral replication [1]. Since the discovery that tropism is determined by capsid surface proteins, mosaic vectors have been constructed to genetically modify viral capsids to create chimeric virions with intergenotypic domain and amino acid substitutions [61].

When AAV infects a cell alone, it cannot replicate autonomously and results in latent infection as double-stranded circular DNA, episome, or by integrating into the chromosome. On the other hand, when helper viruses such as adenoviruses and herpesviruses coexist, the function of these genes, including E1A (early region protein 1A), E1B (early region protein 1B), E2A (early region protein 2A), E4 (early region protein 4), and VA (viral associated), are used to prevent replication of the AAV genome. Similarly, in cultured cells, proliferation occurs only when infection with the helper virus is established. The life cycle of AAV is divided into latent infection and lytic infection. In the former, when infected alone, it integrates into the AAVS1 region of the long arm of chromosome 19 (19q13.3qter) of the host cell at a low integration rate. This integration is due to non-homologous recombination where Rep78/Rep68 binds to a base sequence (GAGC repeat sequence) that is common to both the AAVS1 region and the Rep binding region of the ITR [62]. Therefore, when wild-type AAV infects a target cell, Rep binds to both the AAV ITR and AAVS1, and site-specific integration of the AAV genome into chromosome 19 occurs via the Rep protein. When a cell is co-infected with AAV and an adenovirus, AAV replication occurs, and a large amount of virus is released due to cell destruction (lytic infection) [28]. For AAV to re-enter the lytic phase, it must be co-infected with a helper virus that activates AAV genome replication. These viruses include species such as adenovirus and herpes simplex virus [63]. 

Specific adenoviral genes and associated viral proteins, such as E1a, E1b55k, E2a, and E4orf6, confer the ability to replicate known helper functions for AAV. E1a activates the adenoviral E1b55k, E2a, and E4orf6 promoters and related viral proteins and binds to the YY1 repressor to release AAV p5 promoter repression [64]. As a result, p5 promotes massive expression of Rep68/Rep78 [63,64]. E2 has been shown to stimulate AAV replication capacity in vitro, and E1b55k and E4orf6 proteins promote AAV replication and DNA second-strand synthesis [63,65,66]. Viral-associated proteins activate AAV expression by interfering with the phosphorylation of the translation factor EIF2a, and conversely, when phosphorylated, block AAV gene expression [67,68].

## 5. AAV Infection Mechanisms

**Figure 4:**
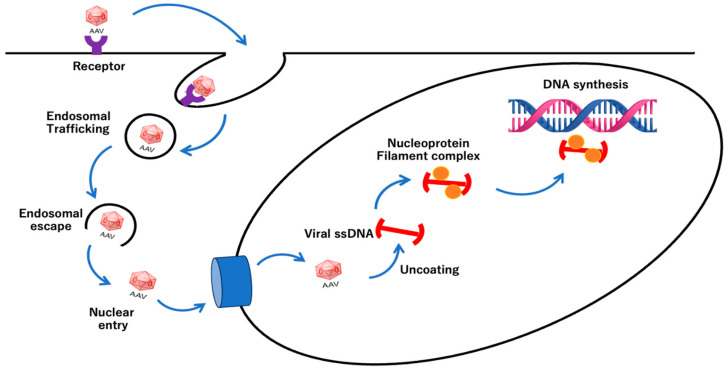
Transduction model of by AAV vectors via binding AAV to cell surface receptor. AAV was internalized into cytoplasm of cell by endosomal trafficking via interaction with AAV receptor (AAVR). The internalized AAVs entry into nucleus through endosomal escape, and then the AAVs within the nucleus release viral single-stranded DNA via uncoating, which forms nucleoprotein filament complex due to transcription.

Viruses are biological species that introduce and amplify their own viral genome into host cells, and AAV also has the property of introducing its own genome into cells [69]. AAV enters human cells via clathrin-coated vesicles that are activated by capsid interactions with receptors located on the outside of the cell surface. The process is mainly divided into the following steps: (1) attachment to the cell membrane; (2) endocytosis (invagination of the plasma membrane); (3) transport by endosomes; (4) escape from the lysosome and late endosome (transport vesicles to the lysosome formed by endocytosis); (5) translocation to the nucleus; (6) formation of double-stranded DNA, which is the replicative form of the AAV genome; (7) expression of the Rep gene; (8) genome replication; (9) expression of the Cap gene and synthesis of progeny single-stranded DNA particles; (10) assembly of complete virus particles; and (11) release from infected cells (Figure 4) [70]. AAV mainly binds to the cell surface using cell surface proteoglycans—a complex of sugar and protein with a special structure, which is a type of complex carbohydrate—as receptors and invades cells [48,71,72,73,74]. The types of proteoglycans recognized when binding to extracellular receptors are those of AAV serum [75]. Once inside the cell, AAV moves along the endocytic pathway [59,76]. AAV can escape degradation by a mechanism called endosomal escape, which is achieved by AAV disassembling the lipid bilayer of endosomes [76,77]. AAV has a phospholipase A (PLA) domain, which is found in the VP1 protein of the AAV capsid and is essential for several functions related to viral escape from endosomes and lysosomes as well as infection processes [13,75,78,79]. Experimental evidence has shown that the sPLA2 domain of murine micro virus is involved in mediating endosomal escape through lipolytic pore formation [80].

In addition, AAV capsid proteins contain a PLA2 domain in the N-terminal regions of VP1, which actively contributes to endosomal escape into the cytosol [80]. The structural change in the PLA domain’s externalization is induced by a decrease in pH as the endosomal pathway progresses [81,82,83]. AAV exits the endosome and translocates to the nucleus of the host cell, releasing its genome [84]. AAV accumulates around the nucleus within a few hours of entry, and its genome is released into the nucleus, where the genome is then replicated [85]. In addition, the transcribed viral-derived mRNA is transported to the cytoplasm where the Rep protein and VP, which plays a role in AAV association and genome packaging, are produced. VP has a nuclear translocation signal on its sequence and moves back from the cytoplasm to the nucleus of the host cell [86,87,88,89]. AAV replicates by assembling new capsids and packaging the replicated viral genome in the nucleus [4,6,68]. In the case of recombinant AAV (rAAV) vectors, in which the encapsulated genome is replaced by the target gene, the route of entry into the cell is the same and the vector enters the nucleus by the same process [89,90,91,92].

## 6. Production of Recombinant AAV (rAAV)

With the development of genetic recombination technology and the biological understanding of wild-type AAV, rAAV has been developed as a gene delivery vehicle in human gene therapy [2]. By replacing the Rep and Cap genes in wild-type AAV with therapeutic genes, the virus does not replicate in the body and can only function as a vector [1,93]. Research studies have shown that AAV vectors used in gene therapy can integrate into the host DNA at a low frequency, typically about 0.1% to 1% of total integrations [19,94]. This integration process is carefully monitored due to concerns about potential genotoxicity and oncogenic transformation, which has led regulatory agencies to require thorough investigation of the safety and efficacy of AAV gene therapy constructs [95,96]. In the absence of a helper virus, the AAV genome does not replicate in infected cells and remains latent in the nucleus as an episome, but in rare cases, it is integrated into the AAVS1 region of the long arm of chromosome 19 through the involvement of the Rep protein at a low integration rate. General rAAV lacks the Rep gene, so site-specific integration into the chromosome does not occur. However, random integration of rAAV into cellular chromosomes can occur at low frequency, and even then, it is likely to insert into actively transcribed gene regions.

Since the tissue tropism of AAV, i.e., whether a given virus can infect certain types of cells, differs among serotypes, it is possible to design vectors that deliver therapeutic genes to specific tissues by selecting an appropriate capsid sequence [97]. rAAV vectors are mainly produced by HEK293 (human embryonic kidney) cells or Sf9 (spodoptera frugiperda) cells [98,99,100]. In the HEK293 cell production method, three types of plasmid DNA are triple transfected into cells, such as a transgene plasmid encoding a target gene inserted between ITR sequences derived from wild-type AAV, a Rep/Cap plasmid encoding the Rep gene derived from wild-type AAV2 and the Cap gene with a specific capsid sequence, and a helper plasmid encoding the proteins necessary for adenovirus-derived AAV production [101,102]. Based on this most widely used calcium phosphate method, HEK293 cells are transfected with three types of plasmid DNA—(i) plasmid with vector sequence, (ii) plasmid with AAV Rep and Cap sequences, and (iii) plasmid with genes corresponding to adenovirus helper function—and the cells are harvested two to three days after transfection and disrupted by freezing and thawing to release vector particles. The standard method is to separate this solution by ultracentrifugation using cesium chloride, purify the vector, and then remove the cesium chloride by dialysis, but various methods such as using ion exchange columns are also being considered for purification [103,104,105,106,107].

There are several AAV purification methods commonly used in the industry to produce clinical-grade AAV vectors. For example, (i) Affinity chromatography: One of the most widely used methods is affinity chromatography, which is based on the specific binding of AAV capsid proteins to immobilized ligands such as antibodies or other affinity resins [108]. This method offers high selectivity and can purify multiple AAV serotypes using specialized affinity resins. (ii) Ion exchange chromatography (IEX): Ion exchange chromatography (IEX) is often used in combination with affinity chromatography as a polishing step [108]. IEX separates AAV particles from contaminants based on surface charge differences, allowing for further purification and removal of empty capsids. (iii) Density gradient ultracentrifugation: Cesium chloride (CsCl) or iodixanol density gradient ultracentrifugation is a traditional method that separates full, partial, and empty AAV capsids based on their density differences [106]. Although effective, it is time-consuming, difficult to scale up, and requires removal of CsCl for clinical use. (iv) Tangential flow filtration (TFF): A novel approach uses tangential flow filtration (TFF) combined with surfactants to inhibit protein aggregation and achieve high clearance of residual proteins and contaminants [109]. (v) Membrane adsorbers: Membrane adsorbers such as ion exchange or hydrophobic interaction membranes can be used for AAV capture and purification, offering advantages such as high binding capacity and scalability [109]. The choice of purification method depends on factors such as AAV serotype, scale of production, and regulatory requirements. Often, a combination of techniques such as affinity chromatography followed by IEX or TFF is used to achieve high purity and yield for clinical-grade AAV vectors [106,108,109].

Since AAV is a virus that uses a helper production mode, its production requires co-infection with an adenovirus as a helper virus [50]. On the other hand, the AAV production method using adenovirus requires a process to remove the adenovirus impurity from the collected cell culture media [110,111]. With this in mind, instead of co-infecting with adenovirus, a method was developed to introduce a helper plasmid, a plasmid that encodes adenovirus-derived proteins necessary for AAV production. There are five proteins required for adenovirus-derived AAV production: E1A, E1B, E2A, E4, and VA (virus-associated RNAs) [1]. When AAV is produced in HEK293 cells, the HEK293 cells naturally produce E1A and E1b, so the helper plasmid encodes three types: E2A, E4, and VA [109,112]. Production using HEK293 cells has the advantage that the production cells are of human origin, and it is easy to construct plasmids for different serotypes [113,114]. In fact, currently approved gene therapies using AAV vectors are produced using HEK293 cells grown by adherent culture [111,115,116]. Suspension culture has also become a standard method for HEK293 production and is widely used by various biopharmaceutical companies for AAV production. In this approach, HEK293 cells are adapted to suspension culture, allowing for scalable and efficient AAV vector production. The process typically involves transient transfection of cells with plasmids to induce AAV production. This method has been optimized for several AAV serotypes (AAV1 to 9) and has shown success in generating high yields of AAV vectors suitable for preclinical and clinical applications [117,118].

However, the production process is complicated, high labor costs are incurred, and scale-up is difficult. Therefore, the current problem is that gene therapy drugs using AAV vectors are extremely expensive [68]. AAV vectors themselves are considered stable reagents and are usually handled from a biosafety perspective, making them easy to handle. On the other hand, the standard amount of vector used is often 1 × 10^4^ or more genome copies per cell in in vitro experiments, and 1 × 10^9^ or more per mouse in various organs including the central nervous system, heart, skeletal muscle, etc., via systemic administration (tail vein, jugular vein, orbital vein, and abdominal vein) and local administration (fixed brain injection, fixed muscle injection, in situ myocardial injection, intravitreal injection, and periarticular injection) [69,110,113,119]. In the AAV production method using Sf9 cells, the target gene and the Rep/Cap gene are introduced into the cells using a recombinant baculovirus as a vector [114,120,121,122]. Since the baculovirus vector itself plays the role of a helper virus, there is no need to introduce additional components necessary for production, such as the helper plasmid used for AAV production in HEK293 cells [123]. Sf9 cells have an established suspension culture system and have the advantage of being easy to scale up. However, disadvantages include the fact that the production cells are not of human origin, that the baculovirus must be removed during purification, and that the stability of the baculovirus vector during cell production is unclear.

In addition, the design-of-experiment (DOE)-optimized protocol for AAV production in suspension cells improved the average yield by 6.1-fold compared to the one-factor-at-a-time (OFAT) method for several AAV serotypes (rAAV1, rAAV2, rAAV5, and rAAV8) [124]. Supplementation of production media with KCl or NaCl increased AAV vector genome yields by 8.5-fold and 7.2-fold, respectively, compared to no salt supplementation [125]. This correlated with a 12-fold and 7.6-fold increase in transducing units (infectious particles), respectively. Typical yields from optimized large-scale (3L) production were around 2–8 × 10^5^ vector genomes per cell [19]. There are different production systems, such as HEK293 suspension; in Sf9 insect cells, stable producer cell lines can give different yields depending on the system [19]. No specific cost data were provided, but the motivation behind optimizing yields and production methods is to reduce the cost of manufacturing AAV vectors [118,124]. Modifying the AAV genome by altering inverted terminal repeats (ITRs) or co-expressing assembly-activating protein (AAP) can improve yields [119]. Reducing contaminants such as empty capsids during upstream production is important for downstream purification [19]. Salt supplementation can specifically improve yields in the herpes simplex virus (HSV) production platform [124]. The DOE approach optimized several factors such as DNA amounts, transfection reagents, and media components for high-yield suspension AAV production [123]. Therefore, the results highlight various strategies such as media optimization, genetic engineering, and process optimization to increase AAV vector yields and reduce manufacturing costs [19,123,124].

## 7. Safety of AAV Vector

**Figure 5:**
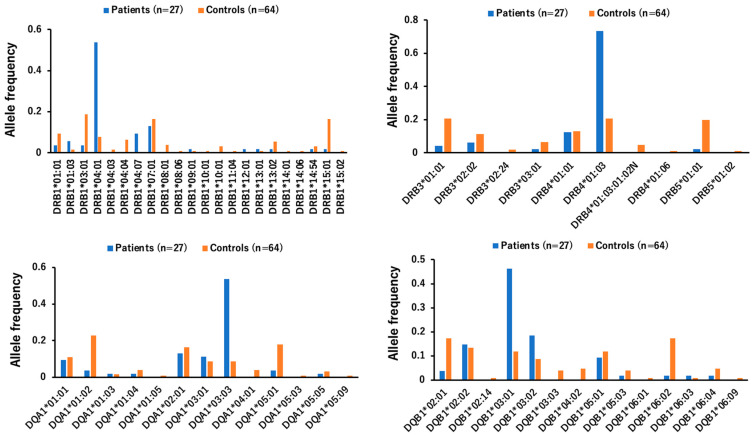
Allele frequency of HLA class II genes [126,131,132].

Infection with wild-type AAV is subclinical and the pathogenicity associated with wild-type AAV infection is unknown. It is also known that integration of wild-type AAV2 into the chromosome is associated with the development of liver cancer [125]. In addition, AAV2 infection was associated with an increase in unexplained childhood hepatitis. In blood samples (14 cases) from children with acute severe hepatitis of unknown cause in the United States, AAV2 was detected in 93% (13 cases) compared to 3.5% (4 cases) in the control group [125,126,127,128]. All 14 children tested positive for human adenovirus. In addition, the patients (13 cases) infected with AAV2 were found to be co-infected with helper viruses that may promote AAV2 replication, such as Epstein–Barr virus or one of the human herpesviruses of the betaherpesvirus HHV-6 group [31,63]. Two UK studies also detected AAV2 in 26 of 32 (81%) and 27 of 28 (96.4%) cases of acute pediatric hepatitis, with lower levels of human adenovirus and human HHV-6B [63,129,130,131,132]. In addition, certain allele frequencies of human leukocyte antigen (HLA) class II genes are increased in the patient group compared to the control group (Figure 5). To ensure the safety and efficacy of AAV vectors in human gene therapy, it is necessary to control the quality of the AAV vectors that serve as gene carriers [109].

There are a variety of quality control points for AAV vectors, including virus titer, structure, contamination rate of empty particles that do not contain the therapeutic gene or incomplete particles that partially contain the therapeutic gene, capsid aggregation, and impurities generated during manufacturing and purification [133]. Not only for AAV vectors, but also for biopharmaceuticals in general, the structure of the protein, which is the main component, is closely related to its function [23,134]. Therefore, it is necessary to properly evaluate the primary structure of the protein, such as amino acid sequences and post-translational modifications, including glycosylation, and the higher-order structure of proteins, such as the folding of the entire capsid structure and the three-dimensional (3D) structure of functional domains, to ensure the efficacy and safety of biopharmaceuticals. Therefore, quality control of AAV vectors requires analytical methods that can be performed at low concentrations and with small amounts of protein. In addition, the degree of post-translational modification varies between AAV production batches [19,64,96,135]. This is because the N-terminus of VP1 and VP-3 is acetylated in several serotypes, including AAV1, AAV2, AAV5, AAV7, AAV9, and AAVrh10, but the N-terminus of VP2 is not [24,136,137]. As described above, it is highly likely that AAV contains variants of the VP component, but the problems are that their analysis has not been sufficiently performed, and furthermore, a method for quantifying the VP ratio has not been established. Therefore, to accurately quantify the VP ratio of AAV vectors for gene therapy, it is necessary to accurately analyze not only VP1, VP2, and VP3, but also all VP-related components contained in the capsid and control their quantitative ratios [138].

In addition, AAV DNA is often found in semen containing mutated sperm, suggesting that it may be involved in male infertility. Research has shown that AAV DNA has been detected in the semen of men with abnormal semen analyses, such as oligoasthenozoospermia or asthenozoospermia, suggesting a possible association between AAV infection and male infertility [138,139]. This suggests a correlation between the presence of AAV DNA in semen and certain sperm abnormalities associated with male infertility.

In addition, the insertion of AAV2 into a specific gene in human hepatocellular carcinoma (HCC) has not been definitively linked to the development of HCC. While the association between AAV2 and HCC is debated, the studies highlight the limitations in establishing a causal relationship between AAV2 and HCC. The presence of AAV2 sequences in HCC tumors is relatively low compared to adjacent normal liver tissue, and there is no clear evidence that full-length AAV genomes are inserted into AAV-positive HCC [140,141,142,143,144]. In addition, epidemiologic data suggest a protective role for AAV infection in patients with cervical cancer. Thus, research suggests that while there are observations regarding AAV2 and HCC, the definitive role of AAV2 in HCC remains uncertain [145,146,147].

The therapeutic use and safety of adeno-associated virus (AAV) vectors are closely related to their structural characteristics and route of administration. Different AAV serotypes (AAV1, AAV2, AAV5, AAV8, and AAV9) have different tissue tropisms and immunogenicity profiles, which influence their safety and efficacy for specific applications [148]. Capsid engineering strategies such as rational design, directed evolution, and machine learning can improve transduction efficiency, reduce off-target effects, and evade neutralizing antibodies, thereby increasing safety and efficacy [149]. Wild-type AAV integrates into the host genome, but recombinant AAV vectors remain mostly episomal, reducing the risk of insertional mutagenesis and genotoxicity [150]. Self-complementary AAV (scAAV) vectors bypass the rate-limiting second strand synthesis step, allowing for faster and more efficient transduction [149]. Genomic modifications such as CpG depletion may reduce innate immune activation and improve safety [149]. Subretinal or intravitreal administration for ocular diseases such as Leber congenital amaurosis and choroideremia is relatively safe, with surgical risks such as macular thinning being the primary concern [148,150]. Intravenous administration in diseases such as hemophilia, muscular dystrophies, and spinal muscular atrophy carries a higher risk of adverse events such as thrombocytopenia, renal injury, and complement activation, especially at high doses (>10^13^ vg/kg) [145,151]. The hepatic tropism of some serotypes can lead to transaminase elevation and potential toxicity [149]. However, some liver transduction may promote tolerogenicity and improve safety. Thus, the therapeutic application and safety of AAV vectors depend on the specific capsid and genome design, as well as the route and dose of administration. Ongoing research aims to develop optimized AAV vectors with improved safety profiles for various clinical applications [149,151].

## 8. Transcription and Serotypes of VP Variants

VP1, VP2, and VP3, which make up the AAV capsid, are produced from a single Cap gene [4,69,91,152,153,154]. The pre-mRNA of the Cap gene undergoes alternative splicing to produce two types: a major mRNA of approximately 2.3 kb and a minor mRNA of approximately 2.6 kb. Although the amount of pre-mRNA varies depending on the time after the Cap gene is introduced into cells, the ratio of major to minor mRNA after alternative splicing is approximately 7:1. Only the minor mRNA retains the VP1 transcription start codon, so the VP1 abundance ratio is relatively low. On the other hand, VP2 and VP3 are translated from the same major mRNA by leaky scanning, a phenomenon in which the start codon is skipped when the ribosome reaches the most upstream translation start site on the mRNA during the translation process in the cytoplasm. RNA viruses use leaky scanning to produce three to four proteins from a single RNA [154,155]. In the case of AAV major mRNA, the VP3 start codon is AUG methionine, while the VP2 start codon is set to ACG, a non-classical start codon. Therefore, the N-terminal amino acid of VP2 is threonine rather than methionine. Thus, ribosomes scanning the large mRNA first reach the upstream transcription start site of VP2, but because the start codon is ACG, a non-classical codon, most ribosomes proceed downstream without initiating transcription, resulting in the abundance ratio of VP2 becoming smaller than that of VP3. Even when translating a small AAV mRNA, it is possible to miss both the VP2 and VP3 start codons and reach the start codon corresponding to the methionine at position 211 in the amino acid sequence. When multiple methionine residues are present near the transcription start site, the Kozak sequence controls the expression levels of VP3 and VP3 variants [22,155]. When multiple methionine residues are present near the transcription start site, the Kozak sequence controls the expression levels of VP3 and VP3 variants [22,155]. When two translatable AUG start codons are placed side by side on a transcript, the start codon with AUG position as +1, −3 position as A, and +4 position as G are dominantly translated in the Kozak sequence, a common sequence that occurs in eukaryotic mRNAs and is primarily involved in the initiation of translation. The VP3 start codon, corresponding to the methionine at position 204 in the amino acid sequence, is A at the −3 position and G at the +4 position [6,156]. On the other hand, the VP3 start codon corresponding to the methionine at position 211 in the amino acid sequence has C at the −3 position and G at the +4 position [6,156]. Therefore, in the Kozak sequence, translation of VP3 is predominant and only relatively small amounts of VP3 variants are produced [26,157]. AAV1, AAV2, AAv3, AAV6, AAV8, AAV10, and AAVrh10 retain a second transcribed methionine residue and are the serotypes from which VP3 variants can be produced [22]. The existence of VP3 variants has been experimentally confirmed in at least the AAV1, AAV2, AAV6, AAV8, and AAVrh10 serotypes, and VP3 variants in these AAV serotypes are likely to be incorporated into the AAV capsid [13,26,156,158]. In AAV5, which does not have a second transcription start site and does not produce VP3 variants, there is no distinct minor peak corresponding to VP3 variants [26,159].

On the other hand, when AAV2 is produced by introducing plasmid DNA with the M203L mutation, which corresponds to the substitution of methionine for leucine at position 203 in the amino acid sequence, introduced into the VP3 sequence region, VP is produced within the cell, but capsids do not associate [160]. Thus, capsids with a high VP3 change rate have a decreased capsid association rate, suggesting that it may destabilize the AAV capsid [13,26,120]. By quantifying the ratio of VP3 variants in AAV vector development for gene therapy and identifying capsids with low content, it is possible to select drug candidates with high production efficiency [161]. The rate of capsid association for AAVs can be reduced by certain factors: The presence of released DNA shows that the release of DNA from AAVs strongly alters the capsid degradation rate, with the degradation rate increasing rapidly as the fraction of complete (DNA-containing) capsids decreases [162]. This suggests that the presence of free DNA released from AAV capsids may decrease the rate of capsid association or assembly. Serotype differences report that different AAV serotypes have different degrees of thermal stability, which is related to capsid dynamics and flexibility [163]. More flexible serotypes, such as AAV2, may have a reduced capsid association rate compared to more rigid serotypes, such as AAV5 [163,164]. While assembly-activating proteins (AAPs) are not essential for capsid assembly of some serotypes, such as AAV5 and AAV11, the absence of AAPs could potentially decrease capsid association rates for serotypes where they play a more critical role [164]. Thus, key factors that may decrease the capsid association rate of AAVs include low pH, the presence of free DNA released from capsids, inherent differences in capsid dynamics between serotypes, and the absence of assembly-activating proteins for certain serotypes [162,163,164].

As a method to reduce VP variants during the AAV production process, it is first necessary to delete DNA sequences that could lead to the translation of unwanted protein components at the time of construction of the Rep/Cap plasmid DNA containing the Cap gene encoding the VP [127,165]. In addition to the classical AUG, other codons that eukaryotic ribosomes can use to initiate translation are ACG and CUG. In cases where a non-classical codon, such as VP2, is intentionally used as the transcription start site, AUG, ACG, and CUG near the transcription start site of each VP can suppress the expression of unexpected VP components by altering the corresponding DNA sequence within a range that does not affect the capsid association rate or the gene transfer rate. In addition, the conditions for introducing each plasmid necessary for AAV production, the cell culture conditions after plasmid introduction, and the purification conditions after AAV capsid production affect the expression level of VP variants [118,119,166]. On the other hand, the amount of helper plasmid introduced, which encodes the adenovirus-derived protein necessary for AAV expression, strongly influences the amount of VP expressed. In addition, host cell-derived proteases can cleave the VP sequence. Therefore, precise control of the amount of helper plasmid introduced into production cells and complete removal of host cell-derived protease during the purification process are ways to prevent contamination of AAV particles with unwanted VP components [167].

## 9. Structural Features and Dynamics of AAV Particles

In AAV vectors, the higher-order structure of the capsid is closely related to its function [16]. The basic structure conserved in AAV of each serotype is an alpha helix (alphaA) and nine beta strands (beta to betaI). In particular, the antiparallel beta barrel structure formed by eight beta strands (betaB to betaI) is called a jelly roll because of its appearance and is the backbone of the VP3 structure. On the other hand, nine loop regions are reported to have different structures depending on the serotype and are called variable regions (VRs). These loop regions are defined as VR1 to VR9 in sequence, and each VR is involved in gene transfer rate, interaction with neutralizing antibodies, binding to heparin sulfate proteoglycans required during cell transfer, etc. Another loop region other than VR is the loop region inserted between betaH and betaI, and the HI loop involved in capsid association is located between betaH and betaI. Therefore, when rotated around the axis, there is a triple symmetry axis, where the structures overlap three times in one rotation, and a quintuple symmetry axis, where the structures overlap five times in one rotation [86,168]. The regions near the VR2 and HI loops form a channel structure with a fivefold symmetry axis, and the loop regions of VR4, VR5, and VR8 form a raised structure with a threefold symmetry axis [86,168]. In particular, the fivefold symmetry axis channel is the pathway through which the PLA domain is externalized during endosomal escape and the viral genome is released from the nucleus, and is a region involved in activity [76,169,170]. Therefore, it is extremely important to properly evaluate the integrity of the AAV vector structure for gene therapy and to ensure the functionality of AAV vectors [69,171]. In addition, evaluation of the higher order structure of gene therapy AAV vectors is essential to ensure their efficacy and safety [1,2,172,173].

## 10. Structurally Dynamic Molecules and Hydrogen/Deuterium Exchange–Mass Spectrometry

Proteins are structurally dynamic molecules that sample different conformations on time scales ranging from bond vibrations on the femtosecond scale to rearrangements of entire protein domains that can occur over many seconds [96,174,175]. These conformational changes are often important aspects of enzyme and protein function. For example, conformational changes induced by ligand binding organize the active site residues required for catalysis, define the substrate binding site in a sequential kinetic mechanism, shield reactive intermediates from the environment, or are important in regulating enzyme function through allosteric networks [176,177]. Conformational dynamics may be conserved during evolution, and perturbations to conserved molecular motions correlate with changes in substrate specificity and the emergence of new enzyme functions [178]. Hydrogen/deuterium exchange–mass spectrometry, in which deuterium is substituted for solvent-exchangeable protons in the protein of interest, is rapidly emerging as a powerful technique for studying how the conformational landscape of proteins responds to perturbations such as ligand binding and mutagenesis [9,179]. The advantages of this approach for elucidating protein structural dynamics are numerous. First, this method can be performed on small amounts of natural proteins or protein complexes in quaternary structural systems [9,179,180]. The enzyme preparation used in the assay does not even need to be highly purified if the bottom-up workflow provides enough reliably identified peptides covering the protein sequence of interest [181]. Furthermore, this method can provide information on conformational dynamics under near-native conditions without the need for site-specific protein labeling as used in single-molecule fluorescence studies [182,183].

In addition, there is no size limit to the proteins or protein complexes that can be studied. Finally, this time-resolved method can be used to study inherently disordered proteins, which are difficult to study with X-ray crystallography. On the other hand, the main limitation of these techniques is that the data are of low structural resolution [180,184]. Although these data can help indicate where steric dynamics are changing and reveal changes in bound conformation, they are less likely to provide much insight into the precise molecular mechanisms driving the observed changes [9,180,181,182,183,184,185]. The deuterium exchange rate—which is a powerful method for biophysical characterization of enzyme conformational changes and enzyme–substrate interactions—at each amino acid residue in a protein is influenced by the higher order structure formed by each residue [186,187]. Thus, this method allows for perturbations to enzyme conformational dynamics to be mapped to the local spatial scale of the peptide, allowing for the assessment of how perturbations alter the dynamics in different regions in the enzyme of interest [182]. The rate of exchange of the amide moiety of the peptide bond is strongly dependent on pH, the local amino acid sequence, and the local structural environment of the amide. Among its many advantages, this method consumes only small amounts of material and can be performed under near-natural conditions without the need for enzyme/substrate labeling [178,179,180]. It can provide spatially resolved information on enzyme conformational dynamics, even for large enzymes and multiprotein complexes [178,182,188,189,190,191]. In regions forming secondary structures, there is a high probability of amide hydrogens from hydrogen bonds with adjacent atoms, and the deuterium exchange rate tends to be low in general [188,189]. Therefore, the deuterium exchange rate of the loop structure is high when it comes to alpha-helices and beta-helices [188]. Amides involved in hydrogen-bonding interactions, such as those present in alpha-helices and beta-sheets, exchange more slowly than amides in unstructured regions of proteins exposed to bulk solvent. The extent of deuterium uptake therefore reflects the structure of the enzyme [192,193]. Enzymes that are conformationally dynamic or that undergo a conformational transition upon ligand binding result in a measurable hydrogen/deuterium exchange response [192,194]. Perturbations in deuterium uptake levels in the presence of different ligands can be used to map ligand binding sites, identify allosteric networks, and understand the role of conformational dynamics in enzyme function [192]. In complete AAV2 particles, peptides distributed mainly in the VP1/VP2 region; the VR2, VR4, HI loop, and VR9 regions show a high deuterium exchange rate. In the complete AAV particle, the unique VP1/VP2 region exists inside the capsid at near room temperature [169].

On the other hand, the VP1/VP2 unique region has high glycine content in its sequence, so it has a high degree of structural freedom and is easily denatured. Therefore, the VP1/VP2 unique region is considered to have a high deuterium exchange rate, even when located inside the capsid. The VR2 and HI loops are regions with a fivefold symmetry axis where the VP1 unique region is externalized during endosomal escape [167,194,195]. Furthermore, considering that the encapsulated genomes of MVM96 and B19 virus97 of the same *Parvoviridae* family are released from a channel with a fivefold symmetry axis, there is also a high possibility that AAV will be adapted fivefold. The high degree of freedom of the fivefold symmetry axis facilitates the passage of the VP1 unique region and the viral genome from the interior of the capsid [169]. VR4 is highly exposed to the outside and is the region that forms the origin of the triple symmetry axis involved in binding to the receptor [44,196,197]. Thus, the high degree of freedom of the threefold symmetry axis contributes to receptor recognition [198]. The tendency for high deuterium exchange rates in these five- and threefold symmetry axes is likely to be a common feature among viruses of the *Parvoviridae* family [32,199]. In addition, the K706A mutant strain of AAV2, which has mutations in the VR9 region, has reduced infectivity compared to the wild type [9,160]. Therefore, the high degree of structural freedom of VR9 may play a role in the ability of AAV2 to infect cells.

## 11. Full and Empty Particles and Secretion of AAV

Comparing the structures of AAV complete and empty particles using cryo-electron microscopy, a slight difference in the capsid surface near the fivefold symmetry axis was observed for AAV2 [20,200,201]. In addition, the deuterium exchange rate tended to be lower for complete particles in general, suggesting that complete particles have lower dynamics in solution than empty particles [180,181,202,203]. The regions where the deuterium exchange rate was significantly low were the region in the VP1-Vp12 unique region, the region including VR1, the region forming the fivefold symmetrical axis channel, and the region near the channel, the region forming the three-fold symmetrical axis protrusion [204]. AAV8, AAv10, and AAVrh39 show that not only the presence or absence of an encapsulated genome, but also the fivefold symmetry axis electrodensity distribution differs between a complete particle and an empty particle. In AAV8 and AAVrh39, an electron density that is not present in the complete particles was confirmed in the inner part of the fivefold symmetry axis of the empty particle [168]. AAVrh39 is a type of AAV that has been studied for its tropism and ability to cross the blood–brain barrier. Research indicates that AAVrh39, together with AAVrh10, has high transduction efficiency in the skin, while both AAVrh39 and AAVrh10 are capable of efferently crossing the blood–brain barrier and transducing neuronal cells. A comparative analysis of different AAV serotypes revealed that AAVrh39 has similar capabilities to AAVrh10 in crossing the blood–brain barrier, suggesting structural and sequence similarities between these two serotypes. The study also highlighted the importance of specific amino acid residues, such as serine 269, in determining the ability to cross the blood–brain barrier [168,205].

Due to the electron density within the fivefold symmetry axis channel and the length of the amino acid residue in the N-terminal region of VP3, which is longer in the empty particle, the VP1/VP2 unique region is externalized through the fivefold symmetry axis channel in the perfect particle and internalized only in the empty particle [168,169]. The VP1/VP2 unique region of many serotypes of AAV has not been confirmed to be completely externalized under physiological conditions, and the general interpretation is that the VP1/VP2 unique region of AAV is located inside the capsid [170]. In addition, perfect particles have a lower degree of freedom in the structure of the VP1/VP2 unique region and around the fivefold symmetry axis [170]. Therefore, there is no structural change that opens the fivefold symmetry axis channel compared to the complete particle, and the VP1/VP2 unique region is also internalized in the perfect particle. Since the complete particle has a genome inside the capsid, the space that the VP1/VP2 unique region can occupy is narrow [206]. As a result of adopting a structure in which the N-terminus of VP3 is pushed to the bottom of the fivefold symmetry axis, the structural dynamics of the VP1/VP2 unique region and the fivefold symmetry axis channel region decrease, and the deuterium exchange rate decreases. As a result, the complete particle of AAV2 has an elongated outer surface along the fivefold symmetry axis compared to the empty particle [207]. Structural changes near VR1 and the triple symmetry axis are due to changes in the dynamics of the local structure around the fivefold diagonal, which leads to a restructuring of the entire capsid structure and induces allosteric structural changes [33,169,208,209].

Like common parvoviruses, AAV is a non-enveloped virus, so it is relatively physicochemically stable, resistant to desiccation, and retains infectivity at room temperature. In general, there are two molecular mechanisms for viral genome release: one occurs upon capsid collapse and the other occurs while the capsid is retained [59,210]. In the case of AAV, the capsid accumulates in the nucleus, so some structural changes in the capsid are required for genome release [8,211,212]. Therefore, elucidation of the conformational changes in the capsid during AAV genome release will lead to an understanding of the molecular mechanism of AAV genome release. Heat-induced AAV release occurs prior to capsid structural collapse [210,212,213]. This AAV genome release is an irreversible reaction, and AAV genome release continues while the capsid state is maintained [214]. This supports a molecular mechanism by which AAV releases its genome while maintaining the capsid. Furthermore, genome release proceeds even at 37 °C, and heat stress at 55 °C is a requirement for accelerated genome release in the body [213,215,216]. In particles after genome release, the regions where the deuterium exchange rate was significantly high were the VP1/VP2 unique region, the N-terminal region of VP3, and the region near the channel of the fivefold symmetry axis [8,217,218]. In the AAV capsid, the fivefold symmetry axis of the VP1/VP2 unique region changes during heating [167]. A structural change occurs in which the VP1/VP2 unique region of the AAV2 complete particle moves to the outside, and the deuterium exchange rate in the VP1/VP2 unique region and the region near the channel of the fivefold symmetry axis increases [168]. The N-terminal region of VP3 that connects to the VP1/VP2 unique region, corresponding to residue numbers 217 to 221 in AAV2, roughly corresponds to the electron density inside the fivefold symmetry axis channel. Since this sequence has an increased degree of structural freedom, externalization of the VP1/VP2 unique region caused a structural change in which the N-terminal region of VP3 also opened outward [155].

Thus, the N-terminal region of VP3, which was located around the fivefold symmetry axis in the complete particle state, undergoes a structural change such that it is exposed to the outside of the capsid when the VP1/VP2 unique region is externalized by heating [168]. This structural transition is observed as high temperatures induce exposure of the VP1u region, leading to unfolding and externalization. The VP1u domain does not contribute significantly to the stability of AAV capsids, as demonstrated by experiments showing no change in thermal stability in the absence of VP1. In addition, a study correlating thermal stability with particle integrity revealed differential stability among AAV serotypes, with AAV2 and AAV8 denaturing at low temperatures compared to AAV1 and AAV5 [75,164]. As a result, the interaction between the N-terminal region of VP3 and the fivefold symmetry axis channel region decreases and the deuterium exchange rate increases. The deuterium exchange rate decreased due to partial folding resulting from a structural change where the fivefold symmetry axis opened [219]. This phenomenon was observed in the context of hydrogen/deuterium exchange–mass spectrometry, a technique that provides insight into the conformational dynamics and function of proteins by measuring the mass changes associated with isotope exchange between amide hydrogens of the protein backbone and its surroundings [181]. The study revealed how changes in protein structure affect the deuterium exchange rate, shedding light on conformational transitions and dynamics within proteins. Comparing the structures of empty and complete particles, the degree of freedom in the structure of the complete VP1/VP2 unique region and the fivefold symmetric axis channel is low [220,221,222]. This is because the contained genome pushed the VP1 and VP2 regions close to the fivefold symmetry axis, and the dynamics of both decreased due to the formation of intermolecular interactions [26,81]. On the other hand, a structural comparison of the whole particle and the particle after genome release shows that the VP1/VP2 unique region, the N-terminal region of VP3, and the fivefold symmetry axis channel region have a high degree of structural freedom in genome release [162,212,214]. This is because heat stress-induced structural changes caused the externalization of the VP1/VP2 unique region and the N-terminal region of VP3, which was located inside the fivefold symmetry channel. As a result, the space inside the fivefold symmetry axis channel becomes vacant and the channel opens, inducing genome release [8,32].

The deuterium exchange rate in a complete particle reflects the secondary and higher-order structure of the capsid, allowing for the dynamics of the capsid structure in solution to be adequately evaluated [202,205]. Furthermore, in the AAV capsid structure, the fivefold symmetry axis channel region and the threefold symmetry axis bulge region have a high degree of structural freedom, which is a common feature of *Parvoviridae* capsid structures [223,224]. In addition, the degree of structural freedom of the VP1/VP2 unique region and the fivefold symmetry axis channel region of the complete particle is lower than that of the empty particle [76]. Due to the presence of the inclusion, the complete particle undergoes a structural change in which the VP1/VP2 unique region, which is also located inside the capsid, is shifted towards the fivefold symmetry axis [32,87,213]. Furthermore, analysis of the physical stability of whole particles against heat stress showed that healing AAV at a temperature of 55 °C can induce irreversible genome release while maintaining the capsid association state. As a result of higher-order structure comparison, the degree of freedom in the structure of the VP1/VP2 unique region, the N-terminal region of VP3, and the fivefold symmetry axis channel region in the particle after genome release increases [167]. This is because a structural change occurs in the entire particle upon heating, in which the VP1/VP2 unique region located inside the capsid and the N-terminal region of VP3 located inside the fivefold symmetry channel are externalized through the fivefold symmetry channel [225]. This structural change that opens the fivefold symmetry axis channel is necessary for genome release [32,216,226]. By evaluating the higher-order structure of AAV vectors in solution, it is possible to distinguish between active particles in terms of higher-order structure, such as empty particles that may be mixed in during the manufacturing process, and particles from which the encapsulated genome has been released [227,228,229].

## 12. Novel AAV Vectors and Tropisms

### 12.1. Screening of Improved AAV Vector

Since AAV vectors have the inherent advantages of long-term expression, low immunogenicity, and low toxicity, and can achieve even higher gene transfer efficiency, a single intravascular administration of AAV vector enables long-term stable gene transfer and high expression in various target organs and target cells [1,230]. Thus, because AAV has excellent characteristics as a viral vector, variants of AAV have been developed. Although clinical research on gene therapy drugs using AAV vectors has progressed rapidly, the structural understanding of the AAV capsid itself is still incomplete and there are still issues to be resolved [215,231]. One of the important considerations in evaluating the therapeutic potential and safety profile of AAV vectors is their distribution and persistence in different organs and tissues [1,58,68]. On the other hand, the use of rational design and directed evolution methods has made it possible to easily create customized AAV vectors with the most appropriate cell tropism [232]. The advantage of this method is that it does not require prior knowledge of the tertiary or quaternary structure of the outer envelope protein of its function. Even if an AAV variant that perfectly matches the desired phenotype is not identified, if a variant close to the desired phenotype is identified, a secondary library of molecular diversity can be generated and AAV variants with a phenotype closer to the desired phenotypes can be identified [233,234,235]. Specifically, this method involves first constructing a large plasmid library of AAV coat protein genes with molecular diversity, then using this plasmid DNA to generate an AAV virus library, which is then used to infect cultured cells or experimental animals [236,237]. After delivery, selective pressure is applied as appropriate, the selected AAV is collected, and the process is repeated by infecting cells or experimental animals again. By repeating this positive selection three to five times, it is possible to identify the coat protein gene that encodes the desired phenotypes. When constructing a highly diverse AAV capsid library consisting of plasmid DNA carrying chimeric AAV genomes consisting of Rep gene and Cap gene variants, variants of the Cap gene can be efficiently constructed using a variety of approaches, including error-prone PCR, random peptide display, DNA family shuffling, or in silico design. Individual capsid variants are cloned in large parallel into AAV vectors to form chimeric AAV genomes, in which each plasmid vector has a one-to-one ratio of normal Rep genes and mutant Cap gene, and a capsid library is constructed for screening [238]. Error-prone PCR uses standard PCR methods to randomly mutagenize the AAV capsid gene [239,240]. This method is performed using a combination of less optimized PCR conditions with a less precise polymerase, longer extension time, higher Mg^2+^ concentration, addition of Mg^2+^, and addition of dNTP concentrations at different concentrations to introduce random point mutations [241].

Random peptide display involves the insertion of random peptide sequences, typically consisting of seven to nine amino acids, into specific sites on the AAV capsid to alter the natural cellular interactions of the virus [240]. Peptides are typically inserted into AAV capsid positions that facilitate surface exposure of the peptide and are also important for virus–host interactions [207,242]. For example, positions 587 and 588 within the VRIII variable region of the AAV2 capsid are suitable insertion sites for most AAV2-based peptide display libraries [242]. This is because the insertion of a peptide into this region disables the heparan sulfate proteoglycan (HSPG), the primary AAV2 receptor motif of AAV2, allowing for the displayed peptide to interact efficiently with cell surface molecules [243,244]. DNA family shuffling is a highly efficient approach to purifying chimeric AAV capsids by molecularly crossing the parental capsid gene derived from different AAV serotypes [235,245,246]. Novel full-length capsid variants can be reconstructed by primer-free PCR, which fragments the parental capsid genes of different AAV serotypes and recombines them based on partial sequence homology [247,248,249]. Alternatively, synthetic shuffling—a combination of rational design, capsid modification based on knowledge of AAV biology, and directed evolution—can be used to generate libraries of high complexity. In this approach, capsid sites suitable for mutagenesis are identified based on detailed structural and sequence analysis of naturally occurring AAV serotypes. Fragments containing the mutations are synthesized and assembled into full-length novel capsid variants. In silico AAV capsid library design utilizes various approaches for computational prediction of capsid variant sequences, contributing to improved AAV performance [1,250]. One commonly used approach is the in silico design of a putative ancestral AAV library, followed by ancestral reconstruction including experimental verification to identify highly potent ancestral capsid sequences with improved affinity [250]. The rationale behind this approach is that evolutionary AAV intermediates that survive the process of natural selection and emerge are likely to have unique properties while maintaining viral structure and function [1]. Machine learning is another commonly used in silico design method that uses computational algorithms to predict the likelihood of viable virus production from hypothetical capsid variants [250]. Machine learning algorithms rely heavily on available input data to learn protein structure–function relationships and apply them to predict the outcomes of complex physiological processes, such as viral capsid assembly [251,252,253,254].

### 12.2. Viral Packaging of Capsid Library

**Figure 6:**
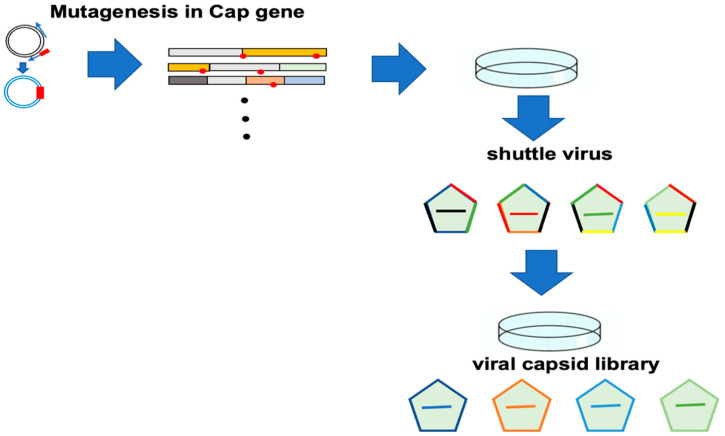
Construction of AAV variant library.

The traditional approach to packaging AAV capsid libraries is a one-step method in which packaging cells are co-transfected with the capsid library and an adenovirus helper plasmid [254]. Although this method is widely used, it has the drawbacks of cross-packaging, the generation of AAV particles whose capsids do not match mutant capsid genomes, and capsid mosaicism, the generation of AAV particles with mosaic capsids derived from capsid proteins from different genomes [255]. To overcome these problems, packaging cells are transfected with a very low plasmid library-to-cell ratio to ensure uptake of a maximum of one single library plasmid per cell. On the other hand, a two-step packaging method first constructs a capsid library by co-transfecting packaging cells with a helper plasmid encoding the wild-type capsid gene but lacking the viral ITR [255,256,257,258]. The Rep and Cap genes of the AAV library plasmid have AAV ITR sequences at both ends and are incorporated into AAV particles as the viral genome [6]. On the other hand, the Rep and Cap genes of the AAV type 2 helper plasmid do not have AAV-ITR sequences, and theoretically, AAV type 2-derived sequences cannot be incorporated into AAV particles as the viral genome [6]. Therefore, although this shuttle virus is a mosaic virus of a mutant capsid protein and an AAV type 2 capsid protein, it does not contain a sequence encoding the AAV type 2 capsid protein, but only the gene encoding the mutant capsid protein. It has the ability to infect HEK293 cells via the AAV type 2 capsid protein, regardless of whether the mutant capsid protein has cell infectivity or not [6,151,259]. This results in the production of AAV particles with a mosaic capsid, called the AAV transfer shuttle, which is partially composed of the wild-type capsid [1,100,203]. The AAV transfer shuttle is then introduced into the packaging cells at a low multiplicity of infection (MOI) to allow for infection by a maximum of one viral particle per cell (Figure 6). The packaging cells are then superinfected with adenovirus, ultimately generating a high-titer viral capsid library whose viral genome information matches the mutant capsid protein. While this two-step method overcomes the problem of mosaicism, it has become clear that this method has a major problem in that the wild-type AAV type 2 contaminates the library. The contaminating wild-type type 2 is produced by homologous recombination between the AAV library plasmid and the AAV type 2 helper plasmid in HEK293 cells [86]. Contamination of a library with wild-type AAV type 2 reduces library diversity and degrades library quality. In addition, because AAV type 2 has a broad host range, repeated positive selection makes AAV type 2 dominant in many target cells, inhibiting the identification of AAV variants with the desired phenotype [260]. To overcome this problem, a method using the AAV type 2 cap gene used in the AAV type helper plasmid has been developed in which the base sequence of the AAV type 2 cap gene is mutated to be as different as possible from the base sequence of the mutant cap gene without changing the amino acid sequence [160].

The use of established cell lines for the selection of AAV capsid libraries is widely used, especially for the identification of AAV variants with altered receptor targeting ability [251,261,262]. Although in vitro selection of AAV libraries is rapid and technically straightforward, there are several challenges [8,214,263]. First, vectors optimized for high transduction efficiency in vitro may not reproduce the same efficiency when used in vivo. Second, AAV vectors that exhibit a high degree of target cell specificity in vitro may transduce non-target tissues when translated in vivo. Another method of selecting AAV libraries in vitro is to expose the library to potent serum from immunized animals before adding it to target cells, specifically to identify variants with immune-evading properties [188]. However, the immune response of AAV variants may change when translated in vivo due to several factors [263,264]. For example, immune recognition of the same AAV vector may change when delivered by different routes [265]. In vivo animal models provide a more reliable platform for screening AAV libraries [1,94]. It is possible to identify AAV variants that can transduce sensitive cell types that cannot be grown in culture, or that can transduce specific cell types with complex tissue structures [38,247,248]. In vivo selection identifies potential off-target effects associated with AAV variants [162,266]. Although both mice and NHPs are widely used for in vivo selection of AAV libraries, the NHP model is the most clinically relevant platform for screening improved AAV vectors [1,267,268].

## 13. AAV-Based Therapies in Clinical Trials

AAV vectors are currently the most widely used for in vivo gene therapy in clinical trials. In terms of AAV serotypes and routes of administration, more than 20 different AAV serotypes/variants are being evaluated in current clinical trials, with AAV2, AAV9, and AAV8 being the most commonly used [268]. Approximately 48% of AAV trials use intravenous injection, which allows for systemic delivery and targeting of organs such as the liver and central nervous system based on vector tropism [269]. Other routes of administration include subretinal, intracranial, intrathecal, intravitreal injections to localize delivery to specific tissues such as eye, brain, etc. [269]. In terms of disease indications, monogenic diseases are the main focus of AAV clinical trials, as AAVs can transduce both dividing and non-dividing cells, allowing for long-term gene expression [269]. Some examples of disease indications in clinical trials are central nervous system disorders such as Parkinson’s disease (AAV2-GAD, AAV2-neurturin, AAV2-AADC), spinal muscular atrophy (AAV9-SMN), and lysosomal storage disorders such as MPS VII (AAV-GUSβ) [33]. In terms of clinical trial results, many AAV trials have shown promising safety and efficacy results, with some resulting in approved gene therapy products [30,269]. For example, the AAV9-SMN trial for spinal muscular atrophy showed significant improvement in motor function in patients with no regression at 2-year follow-up [33]. Thus, AAV vectors, particularly AAV2, AAV9, and AAV8, are the predominant choice for in vivo gene delivery in a wide range of clinical trials targeting monogenic and other diseases, with promising clinical results observed (Table 1). There are five major FDA-approved AAV gene therapies: (i) Luxturna (voretigene neparvovec), the first FDA-approved AAV gene therapy, developed by Spark Therapeutics, approved in 2017 for the treatment of biallelic RPE65 mutation-associated retinal dystrophy, which uses AAV2 vector to deliver functional RPE65 gene to retinal cells [138,269,270,271]. (ii) Zolgensma (onasemnogene abeparvovec), developed by Novartis and approved in 2019 for the treatment of spinal muscular atrophy (SMA) in pediatric patients under 2 years of age, using AAV9 vector to deliver functional SMN1 gene to motor neurons [138,272]. (iii) Hemgenix (etranacogene dezaparvovec), developed by CSL Behring, approved in 2022 for the treatment of hemophilia B in adults, using the AAV5 vector to deliver functional factor IX gene [270,273]. (iv) Elevidys (delandistrogene moxeparvovec), developed by Sarepta Therapeutics, approved in 2023 for the treatment of Duchenne muscular dystrophy (DMD) in patients amenable to exon 53 skipping, using AAVrh74 vector to deliver the microdystrophin gene [270,274]. (v) Roctavian (valoctocogene roxaparvovec), developed by BioMarin and approved in 2023 for the treatment of severe hemophilia A in adults, uses the AAV5 vector to deliver the functional factor VIII gene [270,275]. These AAV gene therapies target a range of genetic diseases by delivering functional copies of defective genes, demonstrating the versatility and potential of this approach.

Key ethical issues related to the use of AAV vectors for gene therapy include the following: (i) Potential for germline transmission: While current AAV gene therapies target somatic (body) cells, there are concerns about inadvertent germline modifications that could be passed on to future generations [276]. This raises ethical questions about consent and implications for offspring. (ii) Long-term safety uncertainties: The potential for latent or delayed adverse effects of AAV vectors is not fully understood because the technology is relatively new [277]. (iii) Access and cost: AAV gene therapies are extremely expensive, raising concerns about equitable access and whether they will be available only to wealthy individuals or populations [278]. (iv) Enhancement and non-therapeutic use: There are ethical debates about the use of AAV for non-therapeutic enhancement of traits such as intelligence or physical ability, rather than for the treatment of disease [69,94]. (v) Public perception and social acceptance: The use of genetic technologies such as AAV could potentially increase intolerance or discrimination against individuals with genetic differences if not properly regulated and ethically implemented. (vi) Regulatory challenges: Establishing appropriate regulatory frameworks and ethical oversight for AAV clinical trials is an ongoing issue given the novelty and uncertainties involved [279]. Thus, key ethical concerns revolve around germline modifications, long-term safety, access and cost issues, potential for enhancement or non-therapeutic use, public perception, and the development of robust regulatory oversight for this emerging technology.

In addition, AAV and lentivirus vectors are two widely used viral vectors for gene delivery, each with their own advantages and disadvantages. Advantages of AAV vectors include (i) non-pathogenicity and low immunogenicity, (ii) ability to transduce dividing and non-dividing cells, (iii) long-term gene expression due to episomal maintenance, (iv) broad tissue tropism with different serotypes, and (v) being relatively stable and easy to produce at high titers [280]. Disadvantages of AAV vectors are (vi) limited packaging capacity (up to ~4.7 kb), (vii) potential for pre-existing neutralizing antibodies in some patients, (viii) they require helper viruses for production, and (ix) inefficient transduction of certain cell types. On the other hand, advantages of lentiviral vectors include (x) the ability to transduce dividing and non-dividing cells, (xi) stable integration into the host genome for long-term expression, (xii) large packaging capacity (up to ~8 kb), (xiii) broad tissue tropism with pseudotyping, and (xiv) efficient transduction of various cell types [281]. Disadvantages of lentiviral vectors are (xv) potential for insertional mutagenesis and oncogenesis, (xvi) biosafety concerns due to HIV-derived backbone, (xvii) potential for silencing of transgene expression, and (xviii) complex production process. AAV vectors are generally considered safer due to their non-pathogenic nature and low immunogenicity, but they have limited packaging capacity. Lentiviral vectors can accommodate larger transgenes and integrate into the host genome for stable long-term expression, but they carry potential risks of insertional mutagenesis and biosafety concerns [282]. The choice between AAV and lentiviral vectors depends on the specific application, target cells/tissues, transgene size, and safety considerations. AAV vectors may be preferred for in vivo gene therapy applications, while lentiviral vectors are commonly used for ex vivo gene delivery and cell therapy applications.

The AAV approach is a promising delivery method for CRISPR (clustered regularly interspaced short palindromic repeat)/Cas systems, offering several advantages but also facing some limitations. The advantages of AAV for CRISPR/Cas delivery include the following: (i) In vivo applicability: AAV vectors can efficiently deliver CRISPR components to various tissues and organs in living organisms, enabling in vivo genome editing. (ii) Safety and low immunogenicity: AAV vectors are generally considered safe and have low immunogenicity, reducing the risk of adverse immune reactions. (iii) Non-integrating nature: AAV vectors remain predominantly episomal, minimizing the risk of insertional mutagenesis. (iv) Efficient delivery of miniature Cas proteins: the limited packaging capacity of AAV can be overcome by using smaller Cas proteins such as SaCas9, CjCas9, Nme1Cas9, Cas12e, and Cas12f, facilitating all-in-one AAV delivery of CRISPR components. (v) Multiplexing capability: AAV vectors can be used to deliver multiple gRNAs simultaneously, enabling multiplex genome editing [281,282]. The limitations of AAV for CRISPR/Cas delivery include the following: (i) Limited packaging capacity: the packaging capacity of AAV vectors is typically less than 5 kb, which limits the size of the CRISPR components that can be delivered. (ii) Production challenges: the production and purification of AAV vectors can be technically challenging and time consuming. (iii) Potential immune response: while AAV vectors have low immunogenicity, pre-existing immunity to AAV in some individuals may limit their efficacy. (iv) tissue tropism: different AAV serotypes have different tropisms for different cell types and tissues, which may limit their applicability in certain contexts. (v) Potential off-target effects: like any genome editing technology, AAV-delivered CRISPR/Cas systems may have off-target effects that need to be carefully evaluated and minimized [276,277]. Therefore, the AAV approach is a promising strategy for delivery of CRISPR/Cas systems, especially for in vivo applications, due to its safety profile, efficient delivery, and compatibility with miniature Cas proteins [281,282]. However, its limitations, such as packaging capacity and production challenges, must be addressed, and potential off-target effects must be carefully monitored and minimized.

## 14. Conclusions

AAV vectors are widely used as vectors that can efficiently deliver genes to target organs by direct in vivo administration and allow for long-term expression. It is common to consider which serotype of vector is best to use depending on the purpose of use, i.e., target cells or target tissues. However, regardless of which serotype vector is used, it is impossible to avoid infection of non-target cells, especially when using AAV type 8 and 9 vectors, which are robust serotypes, regardless of the route of administration. AAV infection can occur in almost all organs of the body. The expression of the introduced genes can be controlled to some extent by transcriptional regulation by tissue-specific promoters. To further increase safety, it is necessary to further improve the efficiency of gene transfer into target cells, reduce the amount of vector administered, and prevent infection of non-target cells. Custom AAV vectors are very important tools to address these issues with current AAV vector systems.

Despite these advantages, challenges remain, including immune responses, potential genotoxicity, limited carrying capacity, and manufacturing complexity, which are areas of ongoing research and optimization. The therapeutic application and safety of AAV vectors depend on the specific capsid and genome design, as well as the route and dose of administration. Ongoing research is aimed at developing optimized AAV vectors with improved safety profiles for various clinical applications.

Affinity chromatography with AAVR and DSF assays enables the purification, quantification, and identity verification of AAV capsids, while hydrogen/deuterium exchange coupled to mass spectrometry provides detailed structural insights to guide rational engineering of AAV vectors for enhanced functionality. It can also map regions involved in AAV–receptor interactions and antibody-binding epitopes, probe conformational changes upon receptor binding or antibody neutralization, characterize structural differences between natural and engineered AAV capsids, and identify stabilizing or destabilizing mutations for rational capsid engineering.

## Figures and Tables

**Table 1:** AAV serotypes, natural tropisms, and clinical trials.

Serotypes	Origin	Primary Receptor	Secondary Receptor	Natural Tropism	Clinical Trials
AAV1	NHP	Sialic acid	AAVR	Muscle, CNS, heart, liver, lung	None
AAV2	Human	HSPG	Integrin, FGFR, HGFR, LamR	Heart, CNS, liver, lung, retina	Pomoe disease, Parkinson’s disease, hemophilia
AAV3	NHP	HSPG	LamR, FGFR, HGFR, AAVR	Liver	None
AAV4	NHP	Sialic acid	Unknown	Retina, lungs, kidney	None
AAV5	Human	Sialic acid	PDGFR, AAVR	Retina, CNS, liver	Hemophilia
AAV6	Human	HSPG, sialic acid	EGFR, AAVR	Heart, liver, muscle, retina	Hemophilia, mucopolysaccharidosis
AAV7	NHP	Unknown	Unknown	Liver	None
AAV8	NHP	Unknown	LamR, AAVR	Muscle, heart, CNS, liver	Eye disease, hemophilia, myopathy
AAV9	Human	Galactose	LamR, AAVR	Heart, CNS, liver	Muscular diseases, pompe disease, Danon disease
AAV10	NHP	Unknown	Unknown	Muscle, myoblast tissue	None
AAV11	NHP	Unknown	Unknown	Muscle, myoblast tissue	None
AAV12	NHP	Unknown	Unknown	Salivary glands, muscle	None

NHP: non-human primate; AAVR: AAV receptor; CNS: central nervous system; HSPG: heparan sulfate proteoglycan; FGFR: fibroblast growth factor receptor; HGFR: hepatocyte growth factor receptor; LamR: laminin receptor; PDGFR: platelet-derived growth factor receptor; EGFR: epidermal growth factor receptor [138].
